# Integrated Analysis of lncRNA-miRNA-mRNA ceRNA Network Identified lncRNA EPB41L4A-AS1 as a Potential Biomarker in Non-small Cell Lung Cancer

**DOI:** 10.3389/fgene.2020.511676

**Published:** 2020-09-18

**Authors:** Meiqi Wang, Sihan Zheng, Xi Li, Yu Ding, Mingyan Zhang, Lin Lin, Hao Xu, Yue Cheng, Xiaonan Zhang, Hui Xu, Shijun Li

**Affiliations:** ^1^Department of Clinical Laboratory, Harbin Medical University Cancer Hospital, Harbin, China; ^2^College of Laboratory Medicine, Dalian Medical University, Dalian, China; ^3^Department of Clinical Laboratory, The First Affiliated Hospital of Dalian Medical University, Dalian, China; ^4^College of Bioinformatics Science and Technology, Harbin Medical University, Harbin, China; ^5^Department of Thoracic Surgery, The Second Affiliated Hospital of Harbin Medical University, Harbin, China

**Keywords:** non-small cell lung cancer, long non-coding RNAs, competing endogenous RNAs, LncRNA-miRNA-mRNA regulatory network, overall survival

## Abstract

**Background:**

Recent evidence has indicated that long non-coding RNAs (lncRNAs) can function as competing endogenous RNAs (ceRNAs) to modulate mRNAs expression by sponging microRNAs (miRNAs). However, the specific mechanism and function of lncRNA-miRNA-mRNA regulatory network in non-small cell lung cancer (NSCLC) remains unclear.

**Materials and Methods:**

We constructed a lung cancer related lncRNA-mRNA network (LCLMN) by integrating differentially expressed genes (DEGs) with miRNA-target interactions. We further performed topological feature analysis and random walk with restart (RWR) analysis of LCLMN. Gene Ontology (GO) and Kyoto Encyclopedia of Genes and Genomes (KEGG) pathway analysis were performed to investigate the target DEGs in LCLMN. The expression levels of significant lncRNAs in NSCLC were validated by quantitative real-time PCR (RT-qPCR). The prognostic value of the potential lncRNA was evaluated by Kaplan-Meier analysis.

**Results:**

A total of 33 lncRNA nodes, 580 mRNA nodes and 2105 edges were identified from LCLMN. Based on functional enrichment analysis and co-expression analysis, lncRNA EPB41L4A-AS1 was demonstrated to be correlated with the tumorigenesis of NSCLC. RT-qPCR results confirmed that the expression levels of lncRNA EPB41L4A-AS1 in NSCLC tissues were downregulated compared with adjacent non-cancerous tissues. Kaplan-Meier analysis showed that high expression of lncRNA EPB41L4A-AS1 was associated with better overall survival (OS) in NSCLC patients. Further investigation identified that high expression levels of COL4A3BP, CDS2, PURA, PDCD6IP, and TMEM245 were also correlated with better OS in NSCLC patients.

**Conclusion:**

In this study, we constructed a lncRNA-miRNA-mRNA ceRNA network to investigate potential prognostic biomarkers for NSCLC. We found that lncRNA EPB41L4A-AS1 could function as a regulator in the pathogenesis of NSCLC.

## Introduction

Lung cancer is the most common cancer for both men and women (11.6% of total cases) and the leading cause of cancer-related death (18.4% of total cancer deaths) in the world (Bray et al., 2018). According to the pathological classification, lung cancer can be classified into small cell lung cancer (SCLC) and non-small cell lung cancer (NSCLC) and approximately 80% of lung cancer cases are non-small cell lung cancer (NSCLC) (Siegel et al., 2019). Despite many improvements developed in diagnostic techniques and treatment strategies, the prognosis of NSCLC is still poor with a 5-year overall survival rate less than 20% (Ramalingam et al., 2011; Sun et al., 2019). As a majority of NSCLC patients are diagnosed at an advanced stage in a majority of patients, it may be the primary reason behind responding to the high mortality rate associated with this disease (Siegel et al., 2019). Therefore, exploring the molecular mechanisms, potential biomarkers for early diagnosis and therapeutic targets of NSCLC remains an important goal for clinic (Wender et al., 2019).

Non-coding RNAs (ncRNAs) used to be regarded ineffective in transcription because they had no function of encoding proteins. Studies of ncRNAs in the past few years had dramatically changed our understanding from useless transcripts to functional regulators which can mediate various cellular processes, including transcription, modification, chromatin remodeling and signal transduction (Cech and Steitz, 2014; Morris and Mattick, 2014; Anastasiadou et al., 2018). As a kind of subclass of ncRNAs, long non-coding RNAs (lncRNAs) are functionally defined as transcripts longer than 200 nt and have key roles in gene regulation (Beermann et al., 2016; Schmitt and Chang, 2016). Increasing evidence had revealed that abnormal expression of lncRNAs were involved cancer development and may serve as prognostic biomarkers and therapeutic targets in cancer (Huarte, 2015; Chi et al., 2018; Kopp and Mendell, 2018).

**FIGURE 1:**
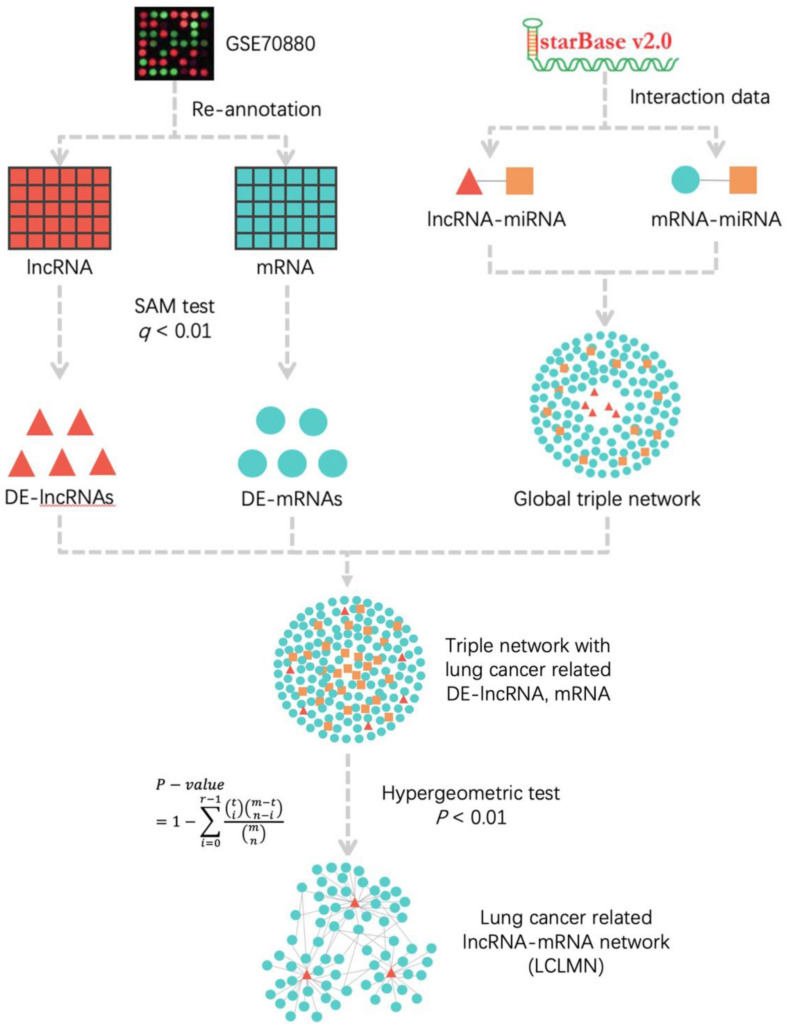
The workflow for construction of lung cancer-related lncRNA-mRNA network. According to the re-annotation results, we used SAM test to calculated the DE-mRNAs and DE-lncRNAs in NSCLC and adjacent non-cancerous samples. The lncRNA-miRNA and mRNA-miRNA interactions were obtained from starBase v2.0 database. We merged all the interactions to construct a global triple network. All the differentially expressed mRNAs and lncRNAs were mapped into a global triple network to extract an expression triple network (DE triple network). Then we combined the significant lncRNA-mRNA interactions to construct LCLMN. lncRNA, long non-coding RNA; miRNA, microRNA; mRNA, messenger RNA; DE-lncRNA, differentially expressed lncRNA; DE-mRNA, differentially expressed mRNA.

Competing endogenous RNAs (ceRNAs) are transcripts that can regulate each other at post-transcription level by competing for shared microRNAs (miRNAs) (Qi et al., 2015). Recent studies reported that lncRNA could interact with miRNAs as a ceRNA network, which is a newly presented model for gene expression regulation. Yang et al. (2019) reported lncRNA LCAT1 was upregulated in lung cancer tissues and serve as an oncogenic gene. LCAT1 may functions as a ceRNA to regulate Rac family small GTPase 1 (Rac1) expression by sponging miR-4715-5p in lung cancer. Another study (Li Y. et al., 2019) demonstrated that lncRNA LINC00641 was downregulated in NSCLC and suppressed cell proliferation and induced cell apoptosis in NSCLC. The LINC00641 may acted as a ceRNA in NSCLC to modulate phospholipid scramblase (PLSCR4) expression levels through miR-424-5p. The ceRNAs are involved in many biological processes and have been found to be an important regulator in many types of cancer including NSCLC (Guo et al., 2015; Wang Q. et al., 2018). However, the function of lncRNAs in the development of NSCLC remains elusive. In this study, we established a lncRNA-miRNA-mRNA ceRNA network to explore the key lncRNAs that can serve as biomarkers in NSCLC (Figure 1). Through topological analysis and random walk with restart (RWR) analysis, we found that lncRNA EPB41L4A-AS1 may function as a potential regulator in the development of NSCLC. We validated the expression levels of lncRNA EPB41L4A-AS1 in 12 paired NSCLC tissues and adjacent non-cancerous tissues by quantitative real-time PCR (RT-qPCR). Moreover, the prognostic value of lncRNA EPB41L4A-AS1 was evaluated in NSCLC by Kaplan-Meier analysis in NSCLC.

## Materials and Methods

### Microarray Data Acquisition

The gene expression profile GSE70880 in this study was extracted from GEO (Gene Expression Omnibus,^1^) database, which contained 20 paired lung cancer and adjacent non-cancerous tissues. The gene expression platform is GPL19748 (Agilent-038314 CBC Homo sapiens lncRNA + mRNA microarray V2.0).

### Probe Re-annotation

The probe sequence related to GSE70880 expression profile is obtained from the GEO database described above. The human mRNA sequence data and lncRNA sequence data are downloaded from GENCODE^2^ database. We used Blastn to identified probe-matched lncRNAs and protein encoded sequences. The length of each probe fragment in the probe target sequences was 60, so we chose 60 as the perfect match length in this study. The ideal alignment results were acquired by the following criteria: (1) Only retained the probes that matched to one transcript, removed the probes matching both the protein-coding transcripts and lncRNA transcripts, leading to two sets of probes-transcripts pairs, respectively; (2) In each set of probes-transcripts pairs, removed the probes matching more than one transcript; (3) Each transcript should be detected matching to more than three probes.

### Identification of Differentially Expressed Genes (DEGs)

Significance Analysis of Microarrays (SAM) is a common method of gene data analysis. It assigns a score to each gene base on the change in gene expression relative to the standard deviation of repeated measurements (Tusher et al., 2001; Shahjaman et al., 2017). Based on the screened data of expression profiles in mRNAs and lncRNAs, we used SAM test to analysis the differentially expressed mRNAs and lncRNAs in lung cancer and adjacent non-cancerous samples. We considered that adjusted *P*-value < 0.01 was statistically significant.

### Extraction of LncRNA-miRNA and mRNA-miRNA Interactions

StarBase v2.0^3^ database is a widely used dataset online tool, which is developed to systematically identify the RNA-RNA and protein-RNA interaction networks from 108 CLIP-Seq data sets (Li et al., 2014). In this study, the lncRNA-miRNA and mRNA-miRNA interactions were downloaded from starBase v2.0 database. We identified 10212 lncRNA-miRNA interaction pairs and 398122 mRNA-miRNA interaction pairs.

### Construction of Lung Cancer Related LncRNA-mRNA Network (LCLMN)

According to the lncRNA-miRNA and mRNA-miRNA interactions data obtained from starBase database, we established a lncRNA-miRNA-mRNA global triple network as the background network to identify gene interactions. All the differentially expressed mRNAs (DEMs) and lncRNAs (DELs) obtained from the above calculations were mapped into a global triple network, and then differential expression (DE) triple network were extracted.

According to DE triple network, the hypergeometric analysis of *P* < 0.01 was used to select the lncRNA-mRNA interaction pairs related to lung cancer. The *P*-value of hypergeometric analysis was defined as follows:

$$P-\text{value}=1-\sum_{i=0}^{r-1}\frac{\left(\begin{array}{c} t\\ i \end{array}\right)\,\left(\begin{array}{c} m-t\\ n-i \end{array}\right)}{\left(\begin{array}{c} m\\ n \end{array}\right)}$$

where *m* represented the total number of miRNAs in starBase database, *t* denoted the number of miRNAs that interacted with the mRNA, *n* was the number of miRNAs that interacted with the lncRNA and *r* represents the number of miRNAs shared between mRNA and lncRNA. The FDR was used for multiple testing corrected and finally obtained the adjusted *P*-value.

Then we combined all these significant lncRNA-mRNA interaction pairs to construct LCLMN. The network was visualized by using Cytoscape^4^.

### Topological Feature Analysis of LCLMN

To screen the significant lncRNAs in the LCLMN network, we further analyzed the topological feature of the LCLMN network on the basis of the node degree, betweenness centrality and closeness centrality. In a given network graph *G* = (*V*, *E*), *V* denotes a set of nodes, *E* denotes a set of edges, and the order of a given node is defined as the number of edges directly connected to the node, which is expressed by Degree(*v*). Then, if there were *k* edges linked to node *v*, the degree of node *v* was calculated as:

Degree⁢(v)=k

Betweenness centrality (BC), defined as the number of shortest paths between all pairs of nodes through a given node in a network, is an effective method to evaluate the centrality of nodes in a network. The BC of node *v* could be described as follows:

$$\text{BC}\,\left(v\right)=\sum_{s\neq v\neq t\in V}\frac{\sigma_{\text{st}}\,\left(v\right)}{\sigma_{\text{st}}}$$

where σ_st_ represented the number of the shortest paths linking node *s* and node *t*, and σ_st_(*v*) was the node count (*v*) from node *s* to node *t*.

Closeness centrality (CC) represented the mean distance from one node to all other nodes in the network, which was calculated as follows:

$$\text{CC}\,\left(v\right)=\frac{1}{\sum_{k=1}^{n}d\,\left(j,v\right)}$$

where *d* (*j*, *v*) represented the shortest distance from *j* to *v*, and *n* denoted the total number of nodes in the network.

### Random Walk With Restart (RWR) on LCLMN

We use random walk to rank the key nodes in LCLMN network. A random walk in network was defined as an iterative walker’s transition from a certain node to a randomly selected neighbor that started from a given node (node *i*). In this study, the random walk we implemented had the ability of restart with probability r in every time step at node *i*. It was defined as follows:

$$p^{t+1}=\left(1-r\right)\,W\,p^{t}+r\,p^{o}$$

where *W* represented the column-normalized adjacency matrix of the network, *p*^*t*^ was a vector whose size is equal to the number of nodes in the network and the *i*-th element holds the probability of being at node *i* at time step *t*.

In this study, eight lung cancer-related genes (CDC25C, TWIST1, HIF1A, DNMT1, PARP1, CDKN1A, CCNB1, and BIRC5) were selected as seed nodes of RWR model. The initial probability vector *p*^0^ of each seed node was set to 1/*n* (where *n* is the number of seed nodes), while other non-seed nodes were set to 0. The *r* stands for probability, which was set to 0.5. When the difference between *p*^t^ and *p*^t +1^ fell below 10^–10^, the update process was completed, and the stable probability *p*∞ was obtained. All candidate lncRNAs could be ranked according to their corresponding probability in *p*∞ and lncRNAs with high scores were considered to be the most likely lncRNAs related to lung cancer. The topological properties of the network were strictly maintained by random sampling without replacement when doing random disturbance. In iterations, the number of times which each lncRNA score was higher than the actual value was recorded as *m*. The statistical significance *p*-value of each lncRNA was calculated by the ratio of *m* to *n*. In our study, we set *n* to 5000 times.

### Functional Enrichment Analysis

To investigate functional enrichment analysis of the differentially expressed genes in LCLMN, Gene Ontology (GO) and Kyoto Encyclopedia of Genes and Genomes (KEGG) pathway analysis were performed using the DAVID Bioinformatics Tool^5^ (Huang da et al., 2009). In this study, *p* < 0.05 was considered statistically significant. The results were visualized with Cytoscape.

### Sample Collection

In this study, 12 pairs of NSCLC tissues and adjacent non-cancerous tissues were obtained from June 2018 to September 2019 at Cancer Hospital of Harbin Medical University. These specimens were used as a validation cohort and separated from the GSE set. The study protocol had been officially approved by the Human Research Ethics Committee of the hospital and conformed to the guidelines set by the Declaration of Helsinki. All samples were stored at −80°C.

### RNA Extraction and Quantitative Real-Time PCR

The expression levels of lncRNA EPB41L4A-AS1, KB-1732A1.1, RP11-390P2.4, RP11-421L21.3 and HOTAIR were assessed by quantitative real-time PCR (qRT-PCR). The oligonucleotide primers used for real-time PCR are listed in Table 1. Total RNA was extracted from 12 paired samples (12 NSCLC tissues and 12 adjacent non-cancerous tissues) using TRIzol^TM^ Reagent (Invitrogen^TM^) according to the manufacturer’s instructions. Then, total RNA was reversed to cDNA using a PrimeScript^TM^ RT Reagent kit (TaKaRa) and cDNAs were quantified by real-time PCR amplification using a SYBR^®^ Premix Ex TaqTM kit (TaKaRa) on Roche 480 qPCR System (Roche Life Science). The relative expression level was determined with the 2^–ΔΔCt^ method and GAPDH was assessed as an internal control. Each sample was performed in triplicate.

**TABLE 1:** Sequences of primers used for qRT-PCR.

**Gene**	**Forward primer (5′-3′)**	**Reverse primer (5′-3′)**
EPB41L4A-AS1	TTTGCTCTGGGTCCTCTGG	CGCTGCCCTTGTTCACG
KB-1732A1.1	CCTTGCCACTCCTCACTCA	GGTCCTCCTCTATGAACCCTGT
RP11-390P2.4	GACCACCTTCAGGGTTGTTATG	CTCCTTGCCCGCACTTC
RP11-421L21.3	GGCGGCGAAAGGTCAGT	TCAGGAACCAGGCTTAGGAA
HOTAIR	GTGAAACCAGCCCTAGCCT	CGTGTAACAGGCAGGTGGA
GAPDH	GAAGGTGAAGGTCGGAGTC	GAAGATGGTGATGGGATTTC

### Kaplan-Meier Analysis

To evaluate the prognostic value of lncRNA EPB41L4A-AS1, we assessed the correlation between lncRNA EPB41L4A-AS1 and the overall survival (OS) in NSCLC patients, breast cancer patients, gastric cancer patients and ovarian cancer patients by online Kaplan-Meier plotter^6^. The probe 225698_at could represent lncRNA EPB41L4A-AS1. The log rank *P*-value and hazard ratio (HR) were identified and displayed on the website.

### Statistical Analysis

The gene expression data of tumor and paired adjacent samples were analyzed by GraphPad Prism 8.0 (GraphPad Prism Software Inc., San Diego, CA, United States) and were expressed as mean ± SD. We compared these two groups by the two-tailed Student’s-test. *P* < 0.05 was considered statistically significant.

## Results

### Identification of Lung Cancer Related Differential Expressed mRNAs and LncRNAs

To identify differentially expressed mRNAs and lncRNAs in lung cancer, we downloaded the lung cancer related expression profile data GSE70880 from the GEO database, including 20 pairs of lung cancer related samples. Then we re-annotated the probes by BLASTn tools and filtration set and eventually screened 11821 pairs of probe-RNAs and 5757 pairs of probe-lncRNAs.

Based on the results of re-annotation, we calculated the 40 matched samples by means of SAM test to identify the differential expressed mRNAs and lncRNAs. Adjusted *P* < 0.01 was considered statistically significant. Finally, we identified 1072 differentially expressed mRNAs and 199 differentially expressed lncRNAs, which were considered as potential regulators in the occurrence and development of lung cancer.

### Construction of Lung Cancer Related LncRNA-mRNA Network

In this study, we downloaded the data of lncRNA-miRNA interactions and mRNA-miRNA interactions from the starBase v2.0 database and merged them to construct a global triple network. There were 15139 nodes in the network, including 277 miRNAs, 1127 lncRNAs, 13735 mRNAs, 10212 miRNA-lncRNA interactions and 398122 miRNA-mRNA interactions.

**FIGURE 2:**
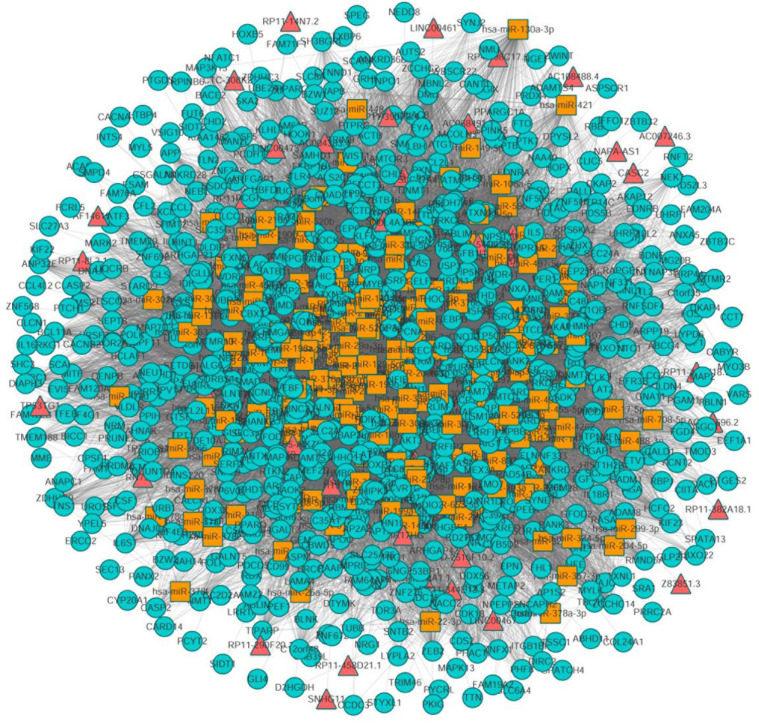
The view of global triple network. The blue, red and yellow nodes represented mRNAs, lncRNAs, and miRNAs, respectively. There were 192 miRNAs, 41 lncRNAs, 775 mRNAs, and 24799 edges in the network.

Next, we mapped 1072 differentially expressed mRNAs and 199 differentially expressed lncRNAs into the global triple network to extract a differential expression (DE) triple network. We considered the lncRNA, miRNA, and mRNA as the nodes of the network. The interactions between these RNAs were defined as the edges of the network. Totally, we obtained 1008 nodes from the network, including 192 miRNAs, 41 lncRNAs, 775 mRNAs, 441 miRNA-lncRNA interactions and 24358 miRNA-mRNA interactions (Figure 2).

**FIGURE 3:**
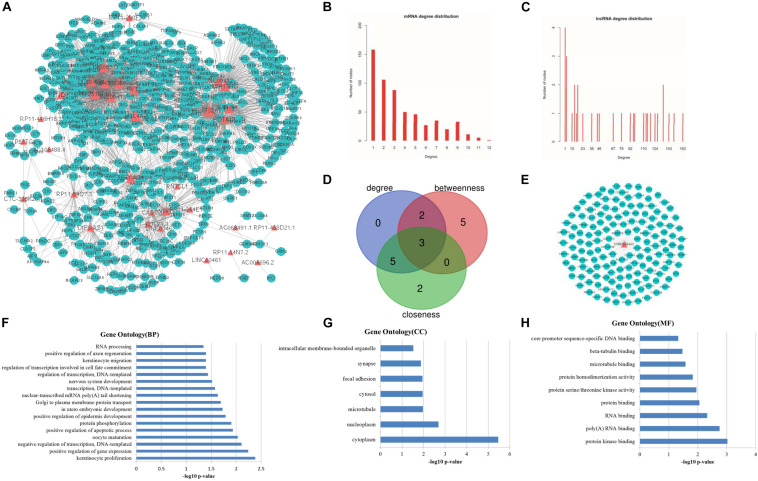
Topological features of LCLMN. **(A)** The overview of LCLMN. The blue nodes represented lung cancer related differential expressed mRNAs and the red nodes represented lung cancer related differential expressed lncRNAs. The size of nodes represented the degrees of the nodes in the network. **(B)** The node distribution of mRNAs in LCLMN. **(C)** The node distribution of lncRNAs in LCLMN. **(D)** The Venn diagram analyses showing the overlap of the top 10 max nodes with topological features in each dimension (degree, betweenness and closeness). **(E)** mRNAs adjacent to lncRNA EPB41L4A-AS1 in LCLMN. **(F–H)** The gene ontology (GO) enrichment analysis of lncRNA EPB41L4A-AS1. The x-axis was the -log10 of *p*-value, and *p* < 0.05 was considered statistically significant. LCLMN, lung cancer-related lncRNA-mRNA network.

To assess the number of lncRNA-mRNA interactions, we screened all candidate lncRNA-mRNA interactions by sharing the common miRNAs. The number of lncRNA-mRNA interaction pairs were obtained by hypergeometric test with adjusted *P*-value < 0.01. Finally, a new lung cancer related lncRNA-mRNA network was established, which contained 33 lncRNA nodes, 580 mRNA nodes and 2105 edges (Figure 3A).

### Topological Analysis of Lung Cancer Related LncRNA-mRNA Network

Topological analysis could screen significant nodes in biological networks, including degree, betweenness and tightness. As shown in Figures 3B,C, we first calculated the degree distribution of mRNAs and lncRNAs in LCLMN. Then we ranked all nodes with topological features from the network and selected top 10 nodes of each dimension. In the overlapping part of these nodes, we found a total of three lncRNAs (EPB41L4A-AS1, KB-1732A1.1, RP11-390P2.4), which might function as crucial regulators in the biological process of lung cancer (Table 2 and Figure 3D). To further clarify the role of these lncRNAs in lung cancer, we analyzed the adjacent mRNAs of these three lncRNAs in LCLMN by GO and KEGG pathway analysis.

**TABLE 2:** The top 10 genes in degree, betweenness, and closeness.

**Gene**	**Degree**	**Gene**	**Betweenness**	**Gene**	**Closeness**
KB-1732A1.1	163	KB-1732A1.1	28553.5564	KB-1732A1.1	0.000112778
EPB41L4A-AS1	150	TPT1-AS1	26549.34041	EPB41L4A-AS1	0.000112727
RP11-390P2.4	143	SNHG14	26246.41373	RP11-390P2.4	0.000112651
AC004383.4	135	EPB41L4A-AS1	18830.89316	AC004383.4	0.000112448
CTD-2516F10.2	135	MIR17HG	18277.10564	CTD-2516F10.2	0.000112397
PVT1	127	LINC00472	16283.77876	SNTB2	0.00011236
MIR17HG	124	HOTAIR	14716.48863	PVT1	0.00011217
LINC00473	118	RP11-54O7.3	13818.89202	LINC00511	0.000112045
LINC00511	114	RP11-390P2.4	12443.66069	LINC00473	0.00011197
LINC00472	113	RP11-421L21.3	12363.29912	NDEL1	0.000111932

*There were three lncRNAs (EPB41L4A-AS1, KB-1732A1.1, and RP11-390P2.4) appeared in each dimension. The node with significant degree, betweenness, and closeness played crucial roles in the network.*

**FIGURE 4:**
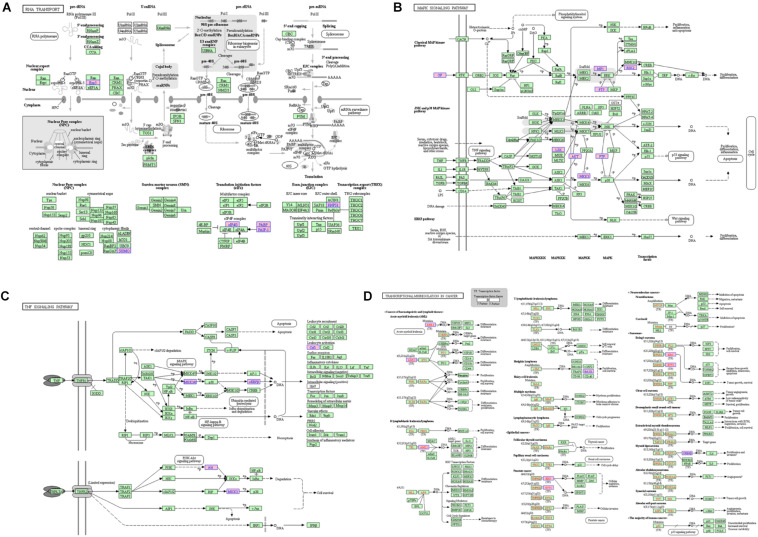
The KEGG pathway related to lncRNA EPB41L4A-AS1. **(A)** RNA transport. **(B)** MAPK signaling pathway. **(C)** Transcriptional misregulation in cancer. **(D)** TNF signaling pathway.

For the lncRNA EPB41L4A-AS1, the adjacent mRNAs in LCLMN was shown in Figure 3E. GO analysis indicated that the differentially expressed genes were mainly enriched in cellular aspects including keratinocyte proliferation, positive regulation of apoptotic process, protein phosphorylation, nuclear-transcribed mRNA poly(A) tail shortening and nervous system development (Figures 3F–H). The results of KEGG pathway analysis showed that these genes involved in four significant cancer-related pathways, such as RNA transport, MAPK signaling pathway, transcriptional misregulation in cancer, and TNF signaling pathway (Figures 4A–D). In recent years, an increasing number of studies had reported that some of these biological processes and pathways were highly associated with lung cancer. As an important intracellular signal transduction pathway, the MAPK signaling pathway controls important cellular biological processes in lung cancer, including transcription, translation, metabolism, apoptosis and cell cycle regulation. Jiang et al. found that hydroxysafflor yellow A (HYSA) can reduce LPS-mediated proliferation, migration, invasion and epithelial-mesenchymal transition (EMT) in non-small cell lung cancer (NSCLC) cells by inhibiting PI3K/AKT/mTOR and ERK/MAPK signaling pathways (Jiang et al., 2019). Xie et al. discovered that miR-148a-3p can induce the inhibition of the proliferation and EMT progression of NSCLC by down-regulating SOS2/MAPK/ERK signaling pathways (Xie et al., 2019). Moreover, Wang et al. reported that resveratrol, as an activator of SIRT1, plays an anti-tumor role in NSCLC by inhibiting Akt/mTOR and activating p38-MAPK pathway (Wang J. et al., 2018). With regard to the other two lncRNAs, lncRNA KB-1732A1.1 and lncRNA RP11-390P2.4, functional enrichment analyses showed that some of the pathways and cell aspects they participated in were also related to the development of some cancers.

**FIGURE 5:**
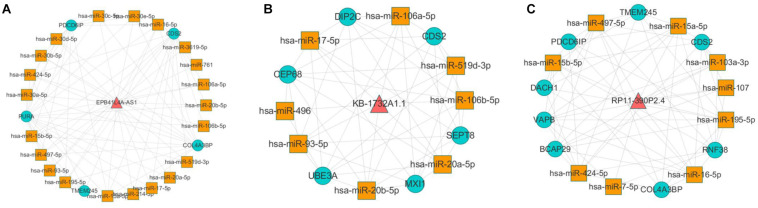
Co-expression analyses of lncRNAs and their neighbor mRNAs in LCLMN. **(A)** The lncRNA EPB41L4A-AS1-miRNA-mRNA network extracted from global triple network. **(B)** The lncRNA KB-1732A1.1-miRNA-mRNA network extracted from global triple network. **(C)** The lncRNA RP11-390P2.4-miRNA-mRNA network extracted from global triple network.

According to the result of topological analysis, we further screened the co-expression of these three lncRNAs and their adjacent mRNA expression matrices in the LCLMN network. The Pearson correlation coefficient (*r*) between lncRNA and mRNA was calculated and 19 lncRNA-mRNA pairs with strong correlations (*r* > 0.6, *p* < 0.05) were selected. Combining with the global triple network, we constructed the ceRNA modules among lncRNAs, co-expressed mRNAs and interacting miRNAs (Figures 5A–C). We discovered that lncRNA EPB41L4A-AS1 could interacted with four miRNAs (miR-761, miR-17-5p, miR-93-5p, miR-106b-5p) (Table 3 and Figure 5A), which were reported to be highly associated with lung cancer (Yan et al., 2015; Wei et al., 2017; Yang et al., 2018; Zhang et al., 2019). For the other two lncRNAs, lncRNA KB-1732A1.1, and lncRNA RP11-390P2.4, the miRNAs of the subnetwork were reported to be involved in the regulation of lung cancer (Li et al., 2017; Li Y. et al., 2019; Yang et al., 2018; Xiao, 2019) (Figures 5B,C). These results indicated that lncRNA EPB41L4A-AS1, lncRNA KB-1732A1.1, and lncRNA RP11-390P2.4 may be implicated in the pathology process of lung cancer. Liao et al. (2019) analyzed the RNA expression profiles of different types of cancer from TCGA and found that the expression of lncRNA EPB41L4A-AS1 was down-regulated in cancer cells. Low expression of lncRNA EPB41L4A-AS1 can reduce the interaction between HDAC2 and NPM1, trigger the Warburg effect, leading to the acceleration of the aerobic glycolysis and glutamine dissolution in cancer cells. Further experiments demonstrated that the expression of lncRNA EPB41L4A-AS1 was associated with poor prognosis. Li J. et al. (2019) found that over-expression of lncRNA KB-1732A1.1 enhanced the growth, migration and invasion of breast cancer cells, and knockdown of lncRNA KB-1732A1.1 significantly inhibited tumorigenesis. Further, in comparison with normal breast tissue in clinical samples, the expression level of lncRNA KB-1732A1.1 in breast cancer patients was often elevated, which showed that lncRNA KB-1732A1.1 plays an important role in regulating EMT and tumor growth, and may be a potential target for breast cancer therapy. Meanwhile, we found no report regarding lncRNA RP11-390P2.4.

**TABLE 3:** lncRNA EPB41L4A-AS1 and co-expressed mRNAs.

**lncRNA**	**mRNA**	**r**	***p*-value**
EPB41L4A-AS1	COL4A3BP	0.616468	2.28E–05
EPB41L4A-AS1	CDS2	0.607038	3.28E–05
EPB41L4A-AS1	PURA	0.602709	3.86E–05
EPB41L4A-AS1	PDCD6IP	0.657878	3.97E–06
EPB41L4A-AS1	TMEM245	0.610812	2.84E–05

### Random Walk With Restart Analysis of Lung Cancer Related LncRNA-mRNA Network

**FIGURE 6:**
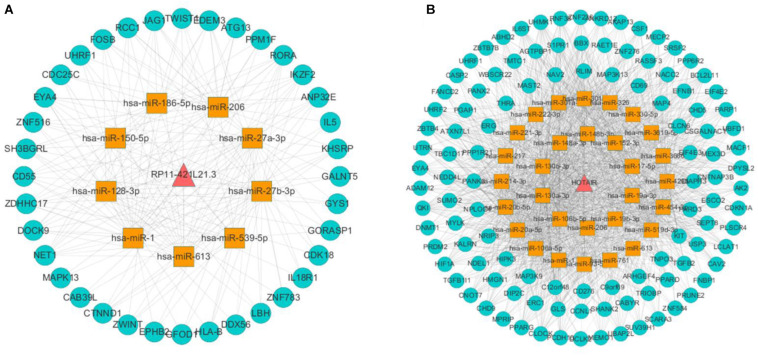
Analysis of NSCLC-related lncRNAs from network and random walk with restart analysis. **(A)** The triple network of lncRNA RP11-421L21.3 extracted from global triple network. **(B)** The triple network of lncRNA HOTAIR extracted from global triple network.

In this study, we obtained eight lung cancer-related genes (CDC25C, TWIST1, HIF1A, DNMT1, PARP1, CDKN1A, CCNB1, and BIRC5) as seed nodes, using an RWR algorithm analysis to prioritize lung cancer related genes. After the 5,000 times random walk with permutation, we identified two lncRNAs (RP11-421L21.3, HOTAIR) with *p* < 0.05. These results indicated that lncRNA RP11-421L21.3 and lncRNA HOTAIR may work as potential regulators in the pathology processes of lung cancer. In addition, to further ascertain the function of these two lncRNAs, according to the lncRNA-miRNA-mRNA model, we extracted their miRNA interaction pairs to construct two sub-networks (Figures 6A,B). We discovered that both lncRNA RP11-421L21.3 and lncRNA HOTAIR could interact with two miRNAs (miR-1 and miR-613), which were showed in the reports of Li J. et al. (2016) and Jiang et al. (2018). In addition, lncRNA HOTAIR was demonstrated to interacted with miR-217 and miR-326. Silencing of lncRNA HOTAIR resulted in over-expression of miR-217, which significantly inhibited the proliferation of NSCLC cells (Chen et al., 2019). Wang R. et al. (2016) demonstrated that through the negative regulation of HOTAIR, mir-326 regulates the proliferation and migration of lung cancer cells by targeting phox2a.

### Validation of LncRNAs in NSCLC

**FIGURE 7:**
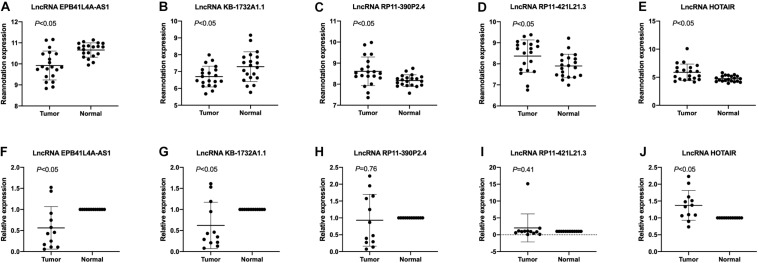
The expression profiles of lncRNA EPB41L4A-AS1, KB-1732A1.1, RP11-390P2.4, RP11-421L21.3, and HOTAIR. **(A–E)** The re-annotation expression level of lncRNA EPB41L4A-AS1, KB-1732A1.1, RP11-390P2.4, RP11-421L21.3, and HOTAIR was evaluated in 20 pairs of NSCLC samples in the microarray. *P*-values were obtained by paired *t*-test (*P* < 0.05). **(F–J)** The expression level of lncRNA EPB41L4A-AS1, KB-1732A1.1, RP11-390P2.4, RP11-421L21.3, and HOTAIR was evaluated by qPCR in 12 pairs of NSCLC samples and corresponding adjacent non-cancerous samples. Data are presented as 2^–ΔΔCt^.

To validate the expression levels of lncRNA EPB41L4A-AS1, KB-1732A1.1, RP11-390P2.4, RP11-421L21.3 and HOTAIR in NSCLC, we performed RT-qPCR on 12 pairs of NSCLC tissues and adjacent non-cancerous tissues. The expression levels of these five lncRNAs in NSCLC tissues were shown in Figure 7. The results confirmed that the expression levels of lncRNA EPB41L4A-AS1 and lncRNA KB-1732A1.1 in NSCLC were significantly lower than that in adjacent non-cancerous tissues, which were consistent with the microarray results (Figures 7A,B,F,G). Both microarray and RT-qPCR results showed that the expression levels of lncRNA HOTAIR were up-regulated in NSCLC tissues (Figures 7E,J), which was consistent with the current studies. For lncRNA RP11-390P2.4 and lncRNA RP11-421L21.3, the re-annotation expression levels were down-regulated in the microarray. However, we did not find any significant difference in the validation experiments (Figures 7C,D,H,I).

### The Prognostic Value of LncRNA EPB41L4A-AS1 in NSCLC

**FIGURE 8:**
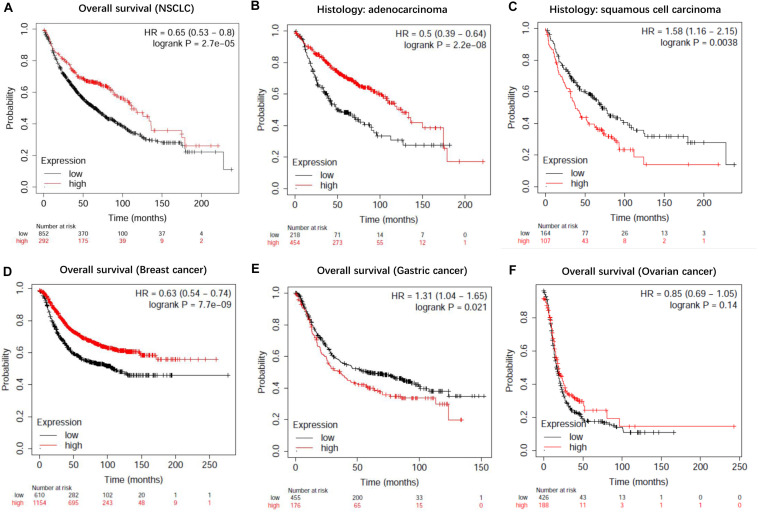
Kaplan-Meier survival curves for lncRNA EPB41L4A-AS1 (225698_at). **(A)** Survival curves were plotted for all NSCLC patients (*n* = 1144). **(B)** Survival curves were plotted for adenocarcinoma patients (*n* = 672). **(C)** Survival curves were plotted for squamous cell carcinoma patients (*n* = 271). **(D)** Survival curves were plotted for gastric cancer patients (*n* = 631). **(E)** Survival curves were plotted for breast cancer patients (*n* = 1764). **(F)** Survival curves were plotted for ovarian cancer patients (*n* = 614). Horizontal axis: overall survival time (months). Vertical axis, survival function; HR, hazard ratio; CI, confidence interval.

**FIGURE 9:**
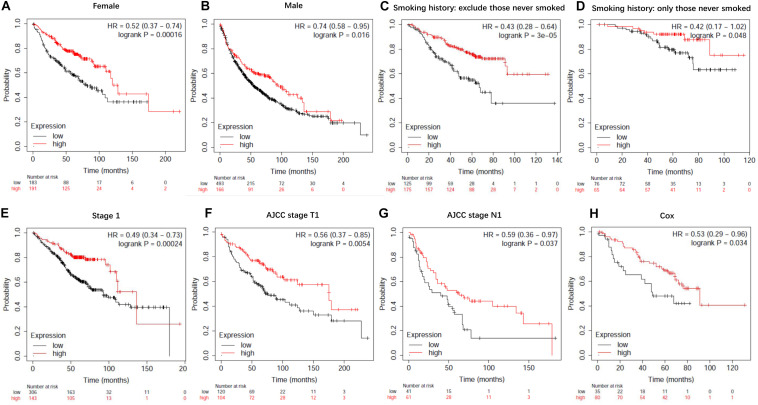
Kaplan-Meier survival curves for lncRNA EPB41L4A-AS1 (225698_at). **(A)** Survival curves were plotted for female patients (*n* = 374). **(B)** Survival curves were plotted for male patients (*n* = 659). **(C)** Survival curves were plotted for all patients excluding who never smoked (*n* = 300). **(D)** Survival curves were plotted for patients never smoked (*n* = 141). **(E)** Survival curves were plotted for patients in stage 1 (*n* = 449). **(F)** Survival curves were plotted for patients in AJCC stage T1 (*n* = 224). **(G)** Survival curves were plotted for patients in AJCC stage N1 (*n* = 102). **(H)** Survival curves were plotted multivariable cox regression analysis (*n* = 115). Horizontal axis, overall survival time (months); Horizontal axis, overall survival time (months); Vertical axis, survival function; HR, hazard ratio; CI, confidence interval.

To evaluate the prognostic value of lncRNA EPB41L4A-AS1 in NSCLC, we performed the Kaplan-Meier analysis of NSCLC patients. Our result showed that high expression of lncRNA EPB41L4A-AS1 was associated with better OS for NSCLC patients (*p* = 2.2e-05) (Figure 8A). Then we probed into the association between lncRNA EPB41L4A-AS1 and other clinicopathological features, including histology, clinical stage, pathological grade, lymph node metastasis status, gender, smoking history, different types of treatments and surgical results. We eventually found that high expression of lncRNA EPB41L4A-AS1 was correlated with better OS in many aspects, such as different types of histology, gender and smoking history (Figures 8B–G). The increasing level of lncRNA EPB41L4A-AS1 was also correlated with better OS in stage 1, AJCC stage T1, AJCC stage N1 and surgical margins negative NSCLC patients (Figures 9A–D).

Then we assessed variables including stage, AJCC stage T, AJCC stage N, gender and smoking history for association with outcome by using multivariable cox regression analysis. According to multivariable analysis results, AJCC stage T was associated with an increased risk of death (hazard ratio [HR] 2.77, 95% CI 1.22–6.26, *P* = 0.015) in lncRNA EPB41L4A-AS1 low expressed adenocarcinoma lung cancer patients.

In addition, we performed Kaplan-Meier analysis in breast cancer patients, gastric cancer patients and ovarian cancer patients to evaluate the prognostic function of lncRNA EPB41L4A-AS1 in other cancers. As a result, high expression of lncRNA RP11-363E7.4 was found to be associated with better OS in breast cancer patients and ovarian cancer patients but for worse OS in gastric cancer patients (Figures 9E,F).

**FIGURE 10:**
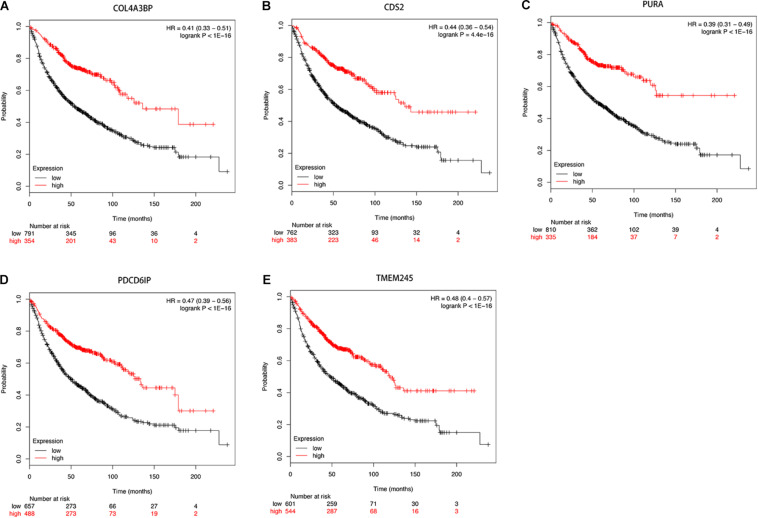
**(A–E)** Kaplan-Meier survival curves for DE-mRNAs correlated to NSCLC patients.

**FIGURE 11:**
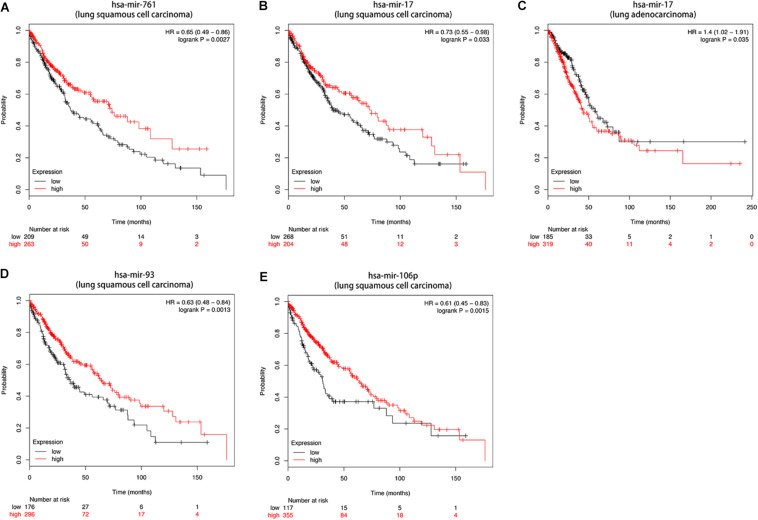
**(A–E)** Kaplan-Meier survival curves for DE-miRNAs correlated to NSCLC patients.

Then we further investigated the association of the co-expressed mRNAs and miRNAs with NSCLC patients’ overall survival to identify the key mRNAs and miRNAs that were related to the prognosis of patients with NSCLC. The results demonstrated that high expression of these mRNAs (COL4A3BP, CDS2, PURA, PDCD6IP, and TMEM245) were all correlated to better OS in NSCLC patients (Figures 10A–E). Moreover, we also discovered that mir-17, mir-761, mir-93, and mir-106p were all correlated to better OS in squamous cell carcinoma patients. It is worth mentioning that high expression of mir-17 in lung adenocarcinoma is associated with worse overall survival (Figures 11A–E).

## Discussion

In this study, we constructed a lung cancer related lncRNA-miRNA-mRNA regulatory network to explore the molecular pathogenesis of non-small cell lung cancer according to the ceRNA hypothesis. To construct LCLMN, we first screened differentially expressed mRNAs and lncRNAs related to lung cancer. Then we generated a global triple network and mapped the differentially expressed genes into it to extract a new subnetwork, containing 33 lncRNA nodes, 580 mRNA nodes and 2105 edges. To further identify the molecular mechanism of NSCLC, we analyzed the topological properties of LCLMN and finally found three important lncRNAs, EPB41L4A-AS1, KB-1732A1.1, and RP11-390P2.4. When performed RWR to the network, lncRNA RP11-421L21.3 and lncRNA HOTAIR were also found in associated with the pathogenesis of NSCLC.

To further investigate the molecular mechanism of NSCLC, we searched the relevant literature and found several reports related to these five lncRNAs (EPB41L4A-AS1, KB-1732A1.1, RP11-390P2.4, RP11-421L21.3, and HOTAIR). LncRNA EPB41L4A-AS1, a p53-regulated gene, has been found to be associated with a variety of human cancers, including breast cancer, colorectal cancer, liver cancer and glioblastomas (Deva Magendhra Rao et al., 2019; He et al., 2019; Liao et al., 2019; Zan and Li, 2019). However, the role of lncRNA EPB41L4A-AS1 in NSCLC has not been fully elucidated. In this study, we validated that the expression levels of lncRNA EPB41L4A-AS1 were decreased in twelve NSCLC tissues compared with adjacent non-cancerous tissues. To further elucidate its function and regulatory pathway, GO analysis and KEGG pathway analysis of lncRNA EPB41L4A-AS1 were performed. The results of GO analysis obtained some crucial biological processes, which are consistent with the current finds of lung cancer development. KEGG pathway analysis showed that lncRNA EPB41L4A-AS1 was mainly involved in four pathways, including RNA transport, MAPK signaling pathway, transcriptional misregulation in cancer, and TNF signaling pathway, suggesting that lncRNA EPB41L4A-AS1 may function as a regulator in the metabolism of NSCLC. Next, combining with the LCLMN network, we further constructed a ceRNA subnetwork among lncRNAs, co-expressed mRNAs and interacting miRNAs. We found that four miRNAs (miR-761, miR-17-5p, miR-93-5p, miR-106b-5p) could interacted with lncRNA EPB41L4A-AS1. Accumulating evidence has indicated that the expression levels of these miRNAs were abnormal in NSCLC tissues, suggesting that they were critical in the tumorigenesis and regulation of NSCLC. Yan et al. (2015) demonstrated that the level of miR-761 detected in serum and tissues of NSCLC patients was significantly up-regulated compared with that of normal participants and paired non-cancerous tissues. Further studies have demonstrated that miR-761 promotes the progression and metastasis of non-small cell lung cancer by targeting ING4 and TIMP2. Zhang et al. (2019) analyzed the expression profiles of several miRNAs in 43 pairs of serum samples and found that the expression of miR-17-5p was significantly up-regulated in patients with NSCLC, suggesting that miR-17-5p has important clinical value in the prediction of NSCLC. Yang et al. (2018) showed that the expression level of miR-93-5p was up-regulated in NSCLC and was significantly associated with the overall survival rate of NSCLC patients. Up-regulated miR-93-5p may function as a tumorigenic factor by inhibiting PTEN and RB1, which may be a new therapeutic target and prognostic indicator for NSCLC. A study conducted by Wei et al. (2017) reported that over-expressed miR-106b-5p promoted cell proliferation and inhibited apoptosis by down-regulating BTG3 expression in NSCLC specimens and cell lines. Furthermore, they found that miR-106b-5p could promote xenograft tumor formation *in vivo*. We found some critical mRNAs co-expressed with lncRNA EPB41L4A-AS1, including COL4A3BP, CDS2, PURA, PDCD6IP, and TMEM245. These protein-coding genes also participated in the regulation of EPB41L4A-AS1-miR-761/miR-17-5p/miR-93-5p/miR-106b-5p interactions. Therefore, we assumed that there were EPB41L4A-AS1-miR-761/miR-17-5p/miR-93-5p/miR-106b -5p-mRNA regulatory networks involved in the regulation of NSCLC tumorigenesis mechanism. However, the specific molecular mechanism of these new regulatory networks still needs experimental validation.

For lncRNA KB-1732A1.1, we found one report on it. Li J. et al. (2019) used microarrays to represent the changing expression of lncRNAs in MCF-7 cells during co-culture of mesenchymal stem cells (MSCS). They found it was up-regulated in breast cancer. Conversely, the expression levels of lncRNA KB-1732A1.1 were down-regulated in NSCLC tissues compared to adjacent non-cancerous tissues in our study. We guess that the role of lncRNA KB-1732A1.1 in the pathogenesis of different types of cancers may be different. Therefore, lncRNA KB-1732A1.1 was not selected as a preferred candidate gene for study.

We found no report regarding lncRNA RP11-390P2.4, hence the expression change and regulating mechanism of lncRNA RP11-390P2.4 in NSCLC is still unclear. Identical to lncRNA RP11-390P2.4, there was no research report on lncRNA RP11-421L21.3. Therefore, based on the ceRNA hypothesis, we further searched for the co-expressed mRNAs and miRNAs associated with lncRNA RP11-421L21.3 and finally discovered several miRNAs (miR-1, miR-128-3p, miR-150-5p, miR-27b-3p, and miR-613), which were correlated with the oncogenesis of NSCLC. Accumulating studies have demonstrated that these miRNAs were down-regulated in NSCLC tissues and could be participated in regulating the proliferation, invasion and metastasis of NSCLC cells through various pathways (Li J. et al., 2016; Chiu et al., 2017; Zheng et al., 2017; Jiang et al., 2018; Pan et al., 2018; Dai et al., 2019; Sun et al., 2019). However, there were no differences in expression levels of lncRNA RP11-390P2.4 and lncRNA RP11-421L21.3 between NSCLC tissues and adjacent non-cancerous tissues in our study. Therefore, we discard these two lncRNAs.

On the basis of previous studies, we found that HOTAIR was up-regulated in many types of cancers, including NSCLC (Zheng et al., 2017; Jiang et al., 2018; Chen et al., 2019). To further investigate the effects of HOTAIR in NSCLC progression, we identified two significant miRNAs, miR217 and miR-326. Chen et al. (2019) showed that in contrast to HOTAIR, the expression of miR-217 was down-regulated in NSCLC cell lines. The silencing of HOTAIR promotes the expression of miR-217 and repressed cell proliferation and migration. Through further experiments, they demonstrated that the up-regulated HOTAIR regulates the tumorigenesis of NSCLC through the miR-217/DACH1 signaling pathway (Chen et al., 2019). Wang R. et al. (2016) demonstrated that silencing of HOTAIR resulted in an increasing expression of miR-326. miR-326 regulates proliferation and migration of NSCLC cells by targeting Phox2a which is regulated by HOTAIR. The functions and ceRNA mechanisms of lncRNA HOTAIR in NSCLC still need to be validated *in vitro* and *in vivo*.

Topological feature analysis and random walk with restart (RWR) analysis of lung cancer related lncRNA-mRNA network are two methods aims to find lung cancer related lncRNAs and the resulting genes can be used for subsequent functional experiments. The lncRNAs determined by topological feature analysis were based on lncRNA-mRNA network and determined by random walk with restart analysis were based on a random walk algorithm. If one lncRNA was identified by both of the two methods, this lncRNA may be considered to have a higher correlation with lung cancer. The lncRNAs obtained by the two methods individually also can be used for subsequent function analysis even if there is no intersection between the two methods.

Although we did not find a lncRNA intersection between the two methods, EPB41L4A-AS1 and HOTAIR were two candidate genes which were found may have association with lung cancer. HOTAIR was a well-known oncogenic lncRNA in human lung cancer. Low expression and deletion of lncRNA EPB41L4A-AS1 were found in a variety of human cancers and associated with poor prognosis of cancer patients, while it was not reported in lung cancer. Therefore, we think the two methods are both effective to explore lung cancer related gene for subsequent function analysis.

Although our study provided an approach to construct a lncRNA−miRNA−mRNA network and finally discovered significant NSCLC-related lncRNAs such as lncRNA EPB41L4A-AS1 and HOTAIR, some limitations should be acknowledged. As the probes used in this study were re-annotated, our network could not cover all lncRNAs, which resulted in some other lncRNAs not being screened out. Our future research will continue to concentrate on expanding the sample size and collecting experimental data to further validate the ceRNA hypothesis.

## Conclusion

In conclusion, we constructed a NSCLC-related ceRNA network and identified five crucial lncRNAs in the network through bioinformatics analysis. It is worth mentioning that lncRNA EPB41L4A-AS1 is closely related to the occurrence and prognosis of NSCLC, which could provide an effective therapeutic target for the treatment of NSCLC in the future.

## Data Availability Statement

The datasets analyzed for this study can be found in the GEO (Gene Expression Omnibus), accession number GSE70880.

## Ethics Statement

The studies involving human participants were reviewed and approved by Harbin Medical University. The patients/participants provided their written informed consent to participate in this study.

## Author Contributions

HuX and SL designed research. MW, SZ, XL, and YD carried out the data collection and data analysis. MZ, LL, and HaX collected clinical samples. MZ, HaX, YC, and XZ performed research. HuX, SL, MW, and SZ wrote the manuscript. All authors read and approved the final manuscript.

## Conflict of Interest

The authors declare that the research was conducted in the absence of any commercial or financial relationships that could be construed as a potential conflict of interest. The reviewer XC declared a shared affiliation, with no collaboration, with several of the authors, SZ, MW, YD, MZ, HaX, YC, XZ, HuX, to the handling editor at the time of review.

## Footnotes

1 http://www.ncbi.nlm.nih.gov/geo/

2 http://www.gencodegenes.org/

3 http://starbase.sysu.edu.cn/

4 http://apps.cytoscape.org

5 http://david.abcc.ncifcrf.gov/

6 https://kmplot.com/analysis/
